# Leaking in Intimate Partner Homicide: A Systematic Review

**DOI:** 10.1177/15248380241237213

**Published:** 2024-03-29

**Authors:** Tanita Rumpf, Stefanie Horn, Catharina Vogt, Kristin Göbel, Thomas Görgen, Kim Marie Zibulski, Vanessa Uttenweiler, Rebecca Bondü

**Affiliations:** 1 Psychologische Hochschule Berlin, Germany; 2 Deutsche Hochschule der Polizei, Germany; 3 Polizeipräsidium Ravensburg, Germany

**Keywords:** domestic violence, predicting domestic violence, homicide, leaking

## Abstract

Intimate partner homicides (IPH) are serious offenses by a heterogeneous group of offenders with diverse risk factors that are too unspecific for the successful prediction of an offense. Recent research suggested several warning signs that may precede IPH and enhance its prevention, but little is still known about “leaking.” Leaking comprises all offense-related statements, behaviors, or actions that express the perpetrator’s thoughts, fantasies, ideas, interests, feelings, intentions, plans, or positive evaluations of an own violent act or previous similar offenses prior to the own attack. This review aims to identify the forms, recipients, and media of leaking as well as potential subgroup differences in cases of IPH. We identified 47 relevant publications via a systematic search of eight databases and additional methods. We included publications that did not explicitly use the term, but described behaviors that could be interpreted as leaking. Up to now, leaking has not been systematically researched in cases of IPH. Nevertheless, publications described several behaviors that are in line with our definition of leaking and were categorized into five broader categories: (a) homicide announcements, (b) previous severe acts of violence, (c) suicidal behavior, (d) planning activities, and (e) interest in similar offenses/offenders. Information on recipients and media as well as subgroup differences was sparse. Leaking is relevant in IPH, but more systematic research is needed to understand its potential role in future risk analyses procedures and prevention of IPH.

Intimate partner homicide (IPH) is a significant public health issue not limited by nationality, social class, or religion (Garcia-Vergara et al., 2022; Graham et al., 2021; Messing et al., 2021; Monckton Smith, 2020): One in seven homicides worldwide is committed by a current or former intimate partner (Stöckl et al., 2013, for a review). Although the majority of all homicide victims are men, according to the Global Study of Homicide, women are the victims of IPH in 82% of the cases (United Nations Office on Drugs and Crime [UNDOC], 2019). Nonetheless, with about 25%, the proportion of female offenders in IPH is exceptionally high as compared to other homicidal offenses (Choromański, 2020, for a case report; UNODC, 2019). Furthermore, there are additional victims, such as family members or new intimate partners, in up to 37% of IPHs (Dobash & Dobash, 2012; Kafka et al., 2021; Smith et al., 2014). Because IPH has stable prevalence rates and an immense impact on bereaved relatives, more research on its prevention is needed (Kapardis et al., 2017; UNODC, 2019). Recent research has suggested that considering leaking, a specific warning sign that points to the preoccupation with or planning of an offense prior to its execution, can be helpful in preventing homicidal offenses in public spaces (Bondü, 2012; Dudenhoefer et al., 2021; Meloy & O’Toole, 2011; Tampe & Bondü, in press). The phenomenon may, therefore, also be helpful to prevent IPH. Thus, the present review aims to give an overview of the current state of research on instances of leaking preceding IPH.

## Intimate Partner Homicide

Contrary to the widespread conception of a crime of passion, IPH mostly does not happen spontaneously, but marks the fatal escalation of a long-term conflict (Dobash et al., 2009; Greuel, 2009; Monckton Smith, 2020). Thus, there may be opportunities for prevention and intervention. Consequently, research has aimed to identify risk factors of IPH: Community and societal risk factors, such as local density of domestic violence, local income rank, or firearm restrictions, have been associated with IPH (Chopra et al., 2022; Zeoli et al., 2018) but do not sufficiently explain which individuals commit IPH. Therefore, it is important to consider individual and partnership-related risk factors or behaviors, such as gun ownership, the presence of children from previous relationships, separation, strangulation, and threats with weapons (Graham et al., 2022; Matias et al., 2020; Smith et al., 2024; Spencer & Stith, 2018 for overviews). Most prominently, a history of intimate partner violence (IPV) preceded 67% to 75% of the IPH cases regardless of the offenders’ sex (Campbell et al., 2007). Nonetheless, these risk factors often lack predictive validity: For example, a history of IPV was not present in all IPH cases and far less than 1% of all IPV cases ended lethally (Boxall et al., 2022; Rye & Angel, 2019). This may explain why even risk assessment tools that were specifically designed to predict IPH (e.g., Danger Assessment by Campbell et al., 2009; Severe Intimate Partner Risk Prediction by Echeburúa et al., 2019; H-Scale by López-Ossorio et al., 2021) fail to accurately predict IPH, especially within proposed categories of extreme danger (i.e., high cutoff scores; Garcia-Vergara et al., 2022; Graham et al., 2021; Thornton, 2017). In addition, these tools are rarely validated and mostly with samples consisting only of male offenders in the U.S. and are, therefore, not transferable to the relatively high proportion of female offenders, from other countries or other atypical offender and victim groups (Graham et al., 2021; UNODC, 2019). This resulted in high false-negative rates (i.e., incorrectly labeling persons as not being at risk of IPH) and low sensitivity of these risk assessment tools. However, most current risk assessment tools were not designed to predict IPH in particular, but IPV reoffending in general (Graham et al., 2021) and, therefore, use common risk factors for IPV. When applied to IPH, this results in high false-positive rates (i.e., incorrectly labeling persons as at risk of IPH) and low specificity of these tools. Thus, to enable effective prevention, new approaches to assess the risk of IPH are required. More specifically, risk markers that are present prior to almost every IPH (high sensitivity) but are otherwise rare (high specificity) would be helpful. One promising risk marker with these characteristics is leaking, the importance of which has been highlighted by research on rare mass-casualty offenses in public spaces.

### Leaking

The concept of leakage was introduced in the context of school shootings as a direct or indirect announcement of an impending violent act (O’Toole, 1999). Research on school shootings or mass murders distinguishes leakage and leaking (Dudenhoefer et al., 2021). *Leakage* comprises any communication about an intended or planned offense to a third party (Meloy & O’Toole, 2011) and is one out of eight potential warnings signs that also include direct threats toward the victim or the identification with previous offenders (Meloy et al., 2012). *Leaking* includes all topic-specific statements, behaviors, or actions by which potential perpetrators reveal their fantasies, thoughts, ideas, intentions, or plans of committing an offense and signal an interest in, a preoccupation with, and a positive evaluation or even the preparation of an offense, similar offenses, or related topics (Bondü, 2012). According to this definition, leaking is the overarching term that includes threats, an interest in prior offenses, preparations of an offense, and other offense-related behaviors. To be considered leaking, the behavior must be potentially observable by third parties who might disapprove of an offense and displayed within a time frame prior to the attack that would still allow for an intervention (Bondü, 2012; Dudenhoefer et al., 2021; Tampe & Bondü, in press).

Leaking is considered a valuable warning sign for severe violence in rare offenses without homogenous offender profiles (Bondü, 2012; Dudenhoefer et al., 2021; Meloy & O’Toole, 2011; Tampe & Bondü, in press): Although leaking is considered to be rare in the general population, it was observed prior to almost all cases of school shootings and terrorist attacks and, therefore, represents a more specific risk indicator than other alleged risk factors (Bondü & Scheithauer, 2014; Tampe & Bondü, in press). It often occurred repeatedly and over long periods of time, that is, already in early stages in the development toward an offense (Bondü, 2012; Niesse et al., 2021; Tampe & Bondü, in press; Verlinden et al., 2000). Additionally, leaking was often communicated to members of the later perpetrators’ family, friends, or colleagues, but also to health care professionals or internet users, thereby creating diverse opportunities to inform law enforcement authorities (Lankford et al., 2019). Because in most cases leaking is not time-sensitive, it leaves room to intervene. Importantly, both direct communications of potential intentions to commit an offense as well as behaviors that reflect an interest in previous offenses, a positive evaluation or justification of such offenses, or a general interest in related topics and weapons, are relevant signals for an impending offense (Tampe & Bondü, in press), particularly if they occur in combination (Bondü, 2012). Because leaking is more frequent than fatal attacks, assessing the risk of an offense should consider its frequency, specific contents, and other criteria. These criteria need to distinguish between individuals who have acted on their leakings (i.e., subsequently committed an offense) and who have not (Bondü, 2012). Two recent risk assessment tools for terrorist attacks based on leaking contents and characteristics were indeed able to discriminate between these two groups, while showing both satisfactory specificity and sensitivity (Niesse et al., 2021; Tampe & Bondü, in press).

Because leaking is an important starting point for the prevention of homicidal offenses in public spaces, it may also be relevant for similar offenses occurring within close relationships, such as IPH. Two studies reported leakage prior to 37% of homicides in close relationships (Greuel, 2009; Meloy et al., 2014). These studies, however, did not include other behaviors that would additionally correspond to the present definition of leaking and suggest a systematic analysis of the available literature. For example, studies mentioned a preoccupation with an offense, planning behaviors, threats, suicidal behavior, verbalized homicidal fantasies, or expressions of sympathy for IPH perpetrators (Boxall et al., 2022; Chopra et al., 2022; Goussinsky & Yassour-Borochowitz, 2012; Monckton Smith, 2020).

Existing risk assessment tools already take some of these behaviors into consideration when aiming to predict the risk for future IPH/IPV, such as death threats, strangulation, or threats with weapons (Campbell et al., 2009; Echeburúa et al., 2009; Garcia-Vergara et al., 2022). As in cases of school shootings and terrorism, however, there may be further forms as well as specific characteristics and contents of leaking that may signal a risk of an impending act and, therefore, could increase the sensitivity and specificity of risk assessment tools. Considering the different aspects of leaking, therefore, is an innovative approach that may help to improve risk assessment and management in relation to IPH. Furthermore, potential victims, their children and loved ones, as well as professionals in touch with them, can be warned by the perception of leaking, life-saving measures can be initiated, and interventions targeting potential perpetrators can take place well in advance.

### The Present Study

This systematic review aims to provide an overview of the current state of research on leaking prior to IPH, regardless of whether the behavior in question was labeled as leaking or otherwise. Specifically, this research investigates the frequency of leaking prior to IPH, its forms, the media used for its communication, its recipients and witnesses, potential subgroup differences (e.g., female vs. male perpetrators), as well as changes over time. The findings will be discussed with regards to existing risk assessment tools, recommendations for future action, and prevention efforts as well as strengths, limitations, and gaps in the existing research.

## Method

### Inclusion Criteria

This review followed the Preferred Reporting Items for Systematic Reviews and Meta-Analyses guidelines (Page et al., 2021). To identify potentially relevant publications, we used a procedure involving multiple stages: We considered descriptive and analytic studies, (multiple) case-studies, and project reports on perpetrators who had deliberately killed or attempted to kill their current or former intimate partners (and additional victims). We only considered studies and reports that contained descriptions of behaviors that were in line with the above presented definition of leaking and were published in German or English between 1999 and 2022. This corresponds to the time period since the introduction of the concept of leaking (O’Toole, 1999). We included intimate relationships between the victim and the perpetrator except for those imagined by the perpetrator and one-time encounters (e.g., one-night stands). We excluded studies that did not provide information specific to IPH (i.e., samples included cases of IPH and homicide of persons other than the partner), that used samples of offenders that were found not guilty by reason of insanity, and that addressed so-called mercy killings.

### Search Strategy

First, we conducted a systematic search on PsycInfo, PsycArticles, Web of Science, PubMed, PubPsych, KrimDok (a German criminological database), and Google Scholar. The search string contained the following keywords combined with Boolean operators “AND” and “OR”: “intimate partner homicide*,” “domestic homicide*,” “spousal homicide*,” uxoricide*, femicide*, androcide*, familicide*, “family annihi*,” leak*, “warning behavi*,” “warning sign*,” threat*, “risk factor*”, plan*, pathway*, “temporal sequencing*,” progression*, stage*, chronolog*, and process*. We searched KrimLit, a second German criminological database, with an adapted search string. Because Google Scholar limits the number of characters per search, we used two separate search strings containing the abovementioned keywords. Due to a multitude of results by Google Scholar, most of which were irrelevant for the present research question, we only included studies that were relevant until we came across 100 subsequent irrelevant publications. Second, we identified articles through hand search and by screening references of relevant publications, particularly meta-analyses and literature reviews.

### Identification

**Figure 1.**
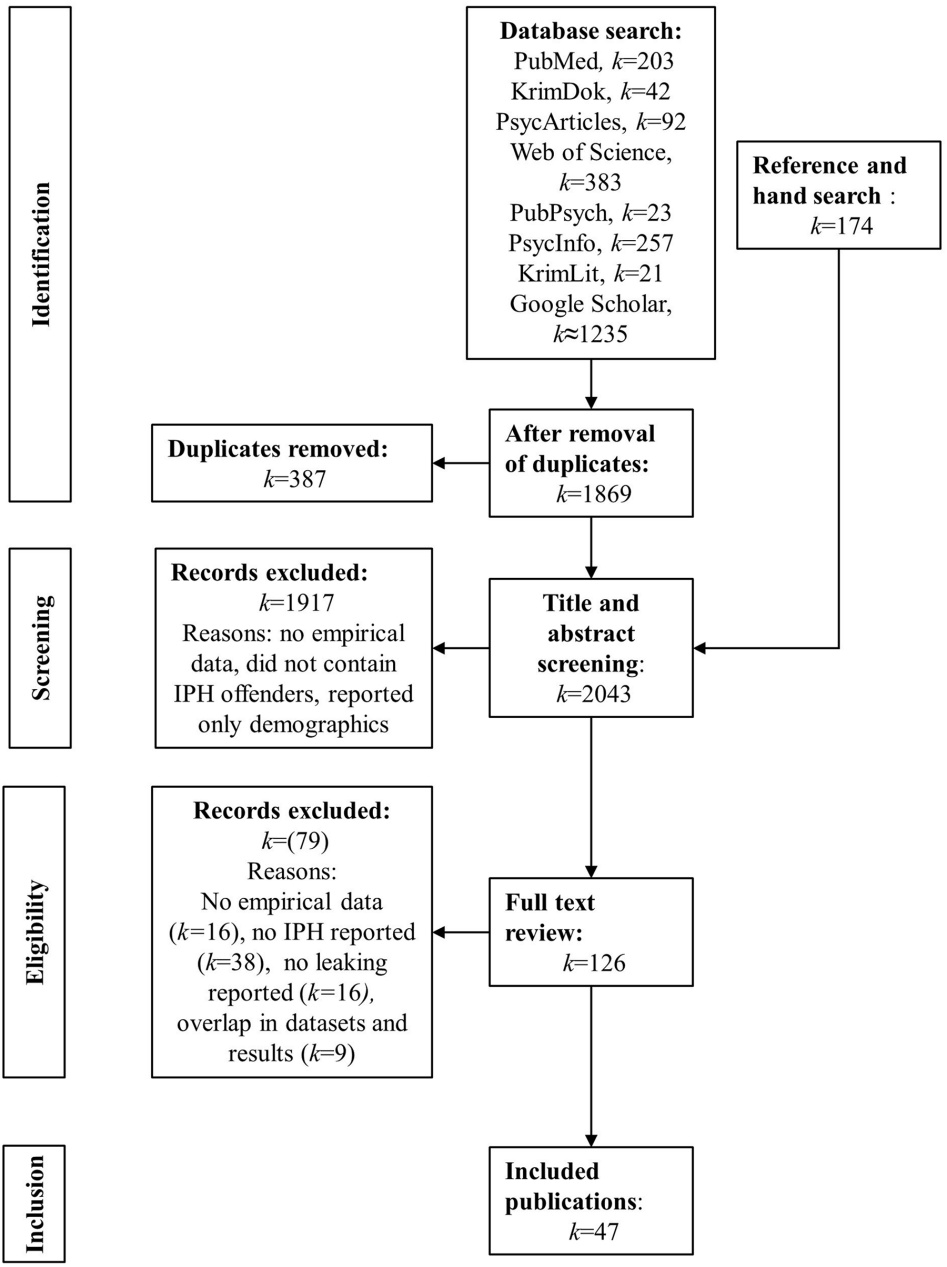
Summary of the identification and inclusion of relevant publications, according to the Preferred Reporting Items for Systematic Reviews and Meta-Analyses procedure, Page et al. (2021).

After adjusting for duplicates (*k* = 387), the search strategy resulted in a total number of 2,043 publications (Figure 1). Two authors independently screened all titles and abstracts with regard to the eligibility criteria by using an online tool (Rathbone et al., 2015). Conflicts in ratings were resolved through discussions between the authors. This resulted in a total number of 126 publications for full-text screening. At least one of the authors screened all of these full-texts. We excluded 79 publications that did not provide empirical data (*k* = 16), specific information on IPH (*k* = 38), or information on leaking (*k* = 16), or used the same dataset as an already included publication without reporting additional information (*k* = 9). This search strategy led to 47 articles for further analysis (marked with * in the reference list).

### Data Extraction

For each publication, we identified the *publication characteristics* (authors, year of publication, publication form [e.g., research report, peer-reviewed article]), the *data collection procedure* (i.e., descriptive or comparative, interviews, retrospective case analysis, analysis of media reports, country, and time span of data collection), the *sample characteristics* (i.e., sample size, percentage of male offenders, offenders’ mean age, and IPH subgroups [male vs. female offenders, familicide, IPH-suicide]), and the information about *leaking*.

## Results

None of the 47 publications used the term “leaking,” but some used related terms, such as “warning signs,” “red flags,” or “alarm bells” (mostly in the abstract or discussion section; Boxall et al., 2022; Kapardis et al., 2017; Musielak et al., 2019; Sharp-Jeffs & Kelly, 2016; Vatnar et al., 2017, 2019). However, most publications did not include a definition of these terms. Hence, our results are based on behavioral examples, designated pathway behaviors, or risk factors that are in line with our definition of leaking (Boxall et al., 2022; Kapardis et al., 2017; Monckton Smith, 2020). Thus, none of the included studies systematically examined leaking in context of IPH.

### Publication Characteristics

Supplementary Material A provides an overview of the 47 included publications. They comprised 40 publications in peer-reviewed journals (85%), three project reports (Boxall et al., 2022; Regan et al., 2007; Sharp-Jeffs & Kelly, 2016), two dissertations (Taylor, 2009; Wiltsey, 2008), one technical report (Monckton Smith, 2016), and one conference paper (Steck, 2005). Of these, 51% (*k* = 24) mainly described risk factors or precursors of IPH, including three single-case (i.e., Anderson et al., 2011; Monckton Smith, 2016; Rabe & Heubrock, 2013) and three multiple-case studies (i.e., Adinkrah, 2000; Glass et al., 2004; Hesselink & Dastile, 2015). Furthermore, 49% of the publications (*k* = 23) compared risk factors of IPH to control groups (e.g., IPV perpetrators; Campbell et al., 2003) or between IPH subgroups (e.g., IPH-suicide; Koziol-McLain et al., 2006). The publications reported results of 51 studies, out of which 48 were included into this review, because the other three did not meet our inclusion criteria (i.e., applied a different definition of IPH or did not report information on perpetrator behavior).

Publications included data that have been collected between 1984 and 2020. Nearly half were conducted in Europe (49%, *k* = 23), 40% in North America (*k* = 19), 6% (*k* = 3) in Oceania, and 2% in Asia (*k* = 1) and Africa (*k* = 1), respectively. Most studies obtained their data through retrospective case-analysis, such as analyzing legal case files or domestic homicide reviews (87%; *k* = 41), and/or interviews (53%; *k* = 25) (of which 72% [*k* = 18] were conducted with bereaved, 28% [*k* = 7] with victims of attempted homicides, 24% [*k* = 6] with perpetrators, and 25% [*k* = 4] with involved professionals). Another 17% relied on news or media reports (*k* = 8). Almost half of the publications used more than one data source (47%, *k* = 22). Fifty-seven percent considered only male perpetrators (*k* = 27), 9% only female perpetrators (k = 4), and 28% both (*k* = 13) (6% reported no information on perpetrator sex [*k* = 3]). Five publications reported results for familicides and six for IPH-suicide perpetrators. The individual publications included 1 offender to 437 offenders with mean ages ranging from 34 to 46 years.

### Forms of Leaking

We identified several behaviors described in the literature as leaking and grouped them into five categories. In the following, we will define each category, name the relevant behaviors, and give further information on frequencies, temporal distance from the impeding attack, as well as further important aspects. Table 1 provides a detailed description.

1. *Homicide Announcements.* Thirty-nine publications reported that in between 16% and 97% of cases, perpetrators expressed their homicidal intentions prior to the offense by verbal statements or threats with weapons. Perpetrators expressed their intentions to kill the victim between 1 day (Boxall et al., 2022) and up to 2 years (Monckton Smith, 2016) prior to the offense. *Verbal expressions or threats to kill the victim* comprise all statements by which the perpetrators explicitly or implicitly revealed an intent to kill the victim. They were reported in 20% to 74% of the cases by 33 publications. In some publications, “threats to kill the victim” were defined as homicide announcements that were communicated directly toward the victim and third parties (Boxall et al., 2022, or studies using risk factors described by the Domestic Violence Death Review Committee, e.g., Cheng & Jaffe, 2021; David & Jaffe, 2021; Jaffe et al., 2014) . *Threats with weapons* include verbal death threats in which using a weapon to kill the partner is described as well as threatening a person with a weapon (e.g., aiming a gun at the victim’s head; frightening the victim with screwdrivers, scissors, or shovels; Cunha & Gonçalves, 2016; Farr, 2002; Glass et al., 2004). Threats with weapons were found in 16% to 67% of the cases and reported in 22 publications.
2. *Previous Potentially Lethal Acts of Violence* (i.e., attempted homicide) and nonfatal, nonsexual strangulation may be considered leaking because they can signal the preparation for or the willingness to engage in fatal violence regarding the specific partner (choking) or intimate partners in general (previous similar acts; Enander et al., 2021; Leygraf, 2015). Whereas the relevance of acts of violence in previous relationships may be subject to debate, their inclusion provides a more comprehensive picture of the potential precursors of IPH. Together, they were reported in 11% to 56% of the IPH cases by 21 publications. Previous attempts to kill an intimate partner were found in 11% to 25% of the cases by 6 publications. Previous strangulation or choking was reported for 11% to 56% of the cases by 18 publications.
3. *Suicidal Behavior*. Disclosures of suicidal intent, preparations for suicide, or suicide attempts that are related to the relationship with the victim were shown 10 days up to 3 years prior to the attack (Anderson et al., 2011; Enander et al., 2021) and reported in 4% to 71% of the cases by 29 publications. Disclosures of suicidal intent (i.e., by suicide thoughts, plans, threats) were reported in 12% to 63% of the cases by 26 publications. Preparations for suicide, such as obtaining or preparing weapons to do so or writing suicide notes were reported in 30% to 39% of the cases by six publications (Boxall et al., 2022; Enander et al., 2021; Logan et al., 2013; Pottinger et al., 2019; Regan et al., 2007; Weeke & Oberwittler, 2017). Attempted suicide was reported in 4% up to 25% of the cases by 16 publications (Cheng & Jaffe, 2021; Hamilton et al., 2013; Pontedeira et al., 2020). Suicidality in context of the intimate relationship can be considered leaking, because it is deeply intertwined with IPH: it functions as a preparation, legitimization or motivation for IPH and 14% to 56% of the perpetrators also killed themselves or had planned to do so (Enander et al., 2021; Glass et al., 2004; Goussinsky & Yassour-Borochowitz, 2012; Sharp-Jeffs & Kelly, 2016; Sheehan et al., 2014).
4. *Planning Activities*. Behaviors that were related to planning, executing, or facilitating an offense were reported in 9% to 75% of the cases by 17 publications and were evident a few hours up to 1 year prior to the impending act (Monckton Smith, 2020). More precisely, drafting homicide plans (e.g., planning to dispose of the victim’s body, an alibi, or to leave the country; stalking or attempting to isolate the victim; researching murder methods; Boxall et al., 2022; Bridger et al., 2017; Monckton Smith, 2020) was reported in 8% to 54% of the cases by 10 publications. Acquiring equipment for the offense was reported in 30% of the cases by six publications. Arming oneself, positioning a weapon at the intended crime scene, or carrying weapons were present in 42% of the cases (Pontedeira et al., 2020) and mentioned by nine publications. Finally, searching for a person to assist the offense was observed in 15% to 75% of the cases by seven publications.
5. *Interest in Similar Offenses/Offenders*. Only one study reported interest in similar offenses within one of the included IPH cases (talking with friends about “wife-killings” on at least two occasions) (Regan et al., 2007).

**Table 1.** Forms of Leaking.

Included Forms	Corresponding Terms/Behaviors	Example	*k*	%
1. Homicide announcements			39	16–97
Statements expressing the intention to kill the victim	Prior threats to kill the victim, threatening the victim’s life, credible threats of death, disclosure of plans or intentions to seriously harm or kill the victim, expression of ideas or thoughts on homicide or serious harm	“If you ever try to leave me like that again, I’ll fucking kill you” (Eriksson et al., 2022, p. 6) “She said, the best thing would be if her husband died, making her his heir” (Enander et al., 2021, p. 7)	33	20–74
Threats with weapons	Use or threat of use of weapons, prior threats with (lethal) weapons, frightening the victim with a weapon	“She threatened the victim with a knife and screwdriver in the year prior to the murder” (Glass et al., 2004, p. 612)	22	16–67
2. Previous severe acts of violence			21	11–56
Attempted homicides	Previous attempts to kill the victim or (attempted) IPH in previous relationships	“She shot her in an incident 5 years previously” (Glass et al., 2004, p. 614)	6	11–25
Strangulation	Nonfatal strangulation, choking (outside the sexual context)	“He put her hands around her throat and strangled her to unconsciousness” (Monckton Smith, 2016, p. 25)	18	11–56
3. Suicidal behavior			29	4–71
Disclosure of suicidal intention	Disclosure, demonstration, or expression of suicide thoughts, ideas, plans, or intentions; suicide threats	“He sent her several text messages saying that he could not live without her” (Todd et al., 2020, p. 8)	26	12–63
Suicide attempts	Prior suicide attempts	“[. . .] he attempted suicide by cutting both his arm lengthwise[. . .]” (Anderson et al., 2011, pp. 158)	16	4–25
Suicide preparations	Suicide notes, suicide preparation	“Had left a suicide note behind” (Pottinger et al., 2019, p. 4)	6	30–39
4. Planning activities			17	9–75
Development of homicide plans	Plotting, (pre-)planning, or preparing the homicide; premediated homicide; research on murder methods; planning the disposal of the victim’s body, an alibi, or how to escape the country	“ [. . .] one female perpetrator prepared the poisoning of her partner [. . .]” (Enander et al., 2021, p. 72)	10	8–54
Obtaining homicide equipment	Purchasing, sourcing, or obtaining weapons, accoutrement, or equipment	“She bought the gun seven days prior to the murder.” (Glass et al., 2004, p. 611)	6	30
Arming oneself with weapons	Putting a weapon in position; taking a weapon to the crime scene, arming oneself with weapons, frequently carrying or walking around with a weapon	“He is an ex-cop that walks around with a pistol in his briefcase” (Sheehan et al., 2014, p. 9)	9	42
Asking for assistance	Plotting the homicide together with a third person; offering another person money to seriously hurt the victim; asking for assistance; instruction or engagement of a third person, a contract killer, or an acquaintance to kill the victim; having an accomplice	“He even offered his brother money to ‘bash’ the victim” (Boxall et al., 2022, p. 38)	7	15–75
5. Interest in similar offenses/offenders	Talking about “wife killings”	“He had had discussed ‘wife murder’ with friends on two occasion” (Regan et al., 2007, p. 24)	1	

*Notes.* Summary of identified forms of leaking including terms found in the literature, descriptive examples, and number of reporting publications (k). Percent ranges are based upon publications that reported frequencies of offenders who displayed the corresponding form of leaking. Single percentages indicate that either only one publication reported frequencies or that at least two publications reported similar frequencies. More detailed information can be found in the Supplemental Material A. IPH = Intimate partner homicides.

### Recipients of Leaking

Studies often did not specify the recipients of leaking (Bridger et al., 2017; Monckton Smith, 2020; Rye & Angel, 2019; Toprak & Ersoy, 2017). Thus, they mostly had to be inferred. The victim was explicitly mentioned as a witness of the perpetrators’ suicidal behavior or death threats by nine publications (Boxall et al., 2022; Enander et al., 2021; Eriksson et al., 2022; Farr, 2002; Goussinsky & Yassour-Borochowitz, 2012; Monckton Smith, 2016, 2020; Taylor, 2009; Todd et al., 2020). The perpetrators’ family, children, friends, acquaintances, and work colleagues were reported to have noticed their intents to kill the victim, suicidality, or search for a contract killer (Anderson et al., 2011; Boxall et al., 2022; Hesselink & Dastile, 2015; Regan et al., 2007; Sheehan et al., 2014). In some cases, general practitioners, psychiatrists, or police officers also knew about the perpetrators’ homicide announcements or suicidality (Monckton Smith, 2020; Regan et al., 2007; Sharp-Jeffs & Kelly, 2016).

### Media of Leaking

No publication systematically analyzed the media through which leaking was communicated. Case examples indicated private conversations, telephone, text messages, killing gestures, written notes, and written plans for the later homicide (Boxall et al., 2022; Farr, 2002; Logan et al., 2013; Monckton Smith, 2020; Sharp-Jeffs & Kelly, 2016; Sheehan et al., 2014; Todd et al., 2020; Weeke & Oberwittler, 2017).

### Subgroup Differences

Publications comparing male and female perpetrators report mixed findings on gender differences in homicide announcements, suicidal behavior, and planning activities, with some suggesting strong overlaps and some suggesting differences between men and women (Bridger et al., 2017; Enander et al., 2021; Leygraf, 2015; Steck, 2005; Weeke & Oberwittler, 2017). Requests for assistance in the offenses were more frequently reported for female (Adinkrah, 2000; Hesselink & Dastile, 2015; Leygraf, 2015; Moen et al., 2016; Steck; 2005) than for male perpetrators. Severe violence—especially nonfatal strangulation—was mostly reported for male perpetrators (Campbell et al., 2003; Jaffe et al., 2014) except for one publication that focused on female perpetrators in homosexual relationships (Glass et al., 2004). Three publications compared IPHs and familicides. Homicide announcements and suicidal behavior did not significantly differ between groups (Jaffe et al., 2014; Hamilton et al., 2013; Weeke & Oberwittler, 2017). Two publications compared cases of IPH with and without the perpetrators’ subsequent suicides. One study reported more frequent pre-offense suicidal behavior and no differences in death threats in IPH-suicide perpetrators as compared to perpetrators without suicide (Koziol-McLain et al., 2006). The other study reported the opposite pattern (Vatnar et al., 2019).

## Discussion

This review highlights the present knowledge about leaking as a specific and valuable warning sign for IPH. Our findings demonstrate a lack of systematic research on the concept in this field. However, frequent reports of behavior that can be considered as leaking, such as expressions of the intent to kill the victim or threats with weapons, as well as a large number of recipients of leaking show that leaking is also relevant in IPH and may aid its prevention (see Table 3 for a summary of key findings).

### Forms of Leaking in Cases of IPH

The five forms of leaking were similar to those in homicidal offenses in public spaces (Bondü, 2012; Dudenhoefer et al., 2021; Fein & Vossekuil, 1998; Tampe & Bondü, in press) and other close relationships (e.g., children as homicide victims; Greuel, 2009); particularly concerning announcements, planning activities, suicidal ideation, and the interest in previous offenses/offenders. However, compared to research on school shootings and terrorist attacks (Bondü, 2012; Meloy & O’Toole, 2011), the included studies did not differentiate between recipients of homicide announcements (e.g., Boxall et al., 2022; David & Jaffe, 2021). Thus, this form does not capture the difference between direct threats (toward the later victim) and *leakage* (announcements communicated toward a third party). Additionally, potentially lethal acts in previous intimate relationships, however, have hardly been relevant in school shooting and terrorist attacks (although some terrorists have attempted or committed several attacks; Tampe & Bondü, in press) and deserve closer research attention. They may indicate a perpetrator’s general willingness to commit IPH corresponding to the present definition of leaking. It may be argued, however, that behavior needs to point to specific offenses and victims to be considered leaking. Thus, it remains to be debated whether potentially lethal acts in previous partner relationships should be considered as leaking or a general risk factor in terms of a stable disposition toward violent behavior.

The different forms of leaking that were described by previous research on IPH may not cover the full spectrum of relevant behaviors. For example, the creation of violent media (e.g., videos, audio files), essays, paintings, and victim lists, an evident interest in weapons, or the identification with previous attackers as described by research on other homicidal offenses (Bondü, 2012; Greuel, 2009; O’Toole, 1999; Tampe & Bondü, in press) were not found. Particularly, an interest in previous offenses and offenders that was important in terrorist attacks (Tampe & Bondü, in press) was only sparsely mentioned in the literature on IPH. This may signal differences in motives: Terrorist attacks often include the orientation toward an existing belief system that allows for the identification with previous offenders as warriors for the true cause; IPH is driven by personal motives related to specific victims (Kivisto, 2015; Meloy, 2001). However, an interest in and compassion for previous offenders (Greuel, 2009; Karlsson et al., 2021) may also have been merely overlooked by most research. Similarly, IPH is often committed with blunt force or weapons at hand (e.g., knives; Matias et al., 2020), making a previous interest in weapons less relevant in cases of IPH as compared to other offenses.

### Media and Recipients

Although the media used for leaking are important, because they may inform practitioners about where to best search for and how to best ask for leaking and because written leaking may provide valuable legal proof for a long term ideation for an offense, only little information about them could be identified.

Supporting previous research (Bondü, 2012; Dudenhoefer et al., 2021), the perpetrators’ family, friends, and work colleagues, as well as police officers and health-care professionals were described as recipients of leaking. Compared to terrorist attacks or school shootings, in which the victims are often unknown to the offenders (Bondü, 2012; Dudenhoefer et al., 2021), leaking in IPH can be expected to be more often directed toward the later victim itself (Greuel, 2009; Meloy, 2001). In these cases, leaking may be used to intimidate and control the later victim. This enables victims to seek help early on (Campbell et al., 2003; Saxton et al., 2022) to try to prevent an offense. In addition, there may be victim behavior that points to an impeding offense to third parties, such as hiding weapons, arming oneself, or taking other precautions (Horn et al., in press; Karlsson et al., 2021; Messing et al., 2015; Sheehan et al., 2014). Thus, future research on leaking in IPH should pay more attention to victim behavior as well.

### Subgroup Differences

This review highlights that leaking is relevant in several IPH subgroups, such as IPH-suicides and familicides as well as male and female offenders (e.g., up to 75% of the female IPH offenders also showed leaking; Adinkrah, 2000). Results, however, were sparse and findings inconsistent, indicating the need for further systematic research. Potential differences in leaking should also be examined between offenses in current and former relationships, because they may have different implications for risk assessment in these cases (Greuel, 2009).

### Criteria to Assess the Seriousness of Leaking

Homicide announcements were found among up to 97% of the IPH perpetrators (Cunha & Gonçalves, 2019) and, thus, the most frequent form of leaking. However, such announcements are by far more common than IPH (Cunha & Gonçalves, 2019; Meloy, 2001; Warren et al., 2008), highlighting the need for further criteria that allow for a reliable assessment of their seriousness. This requires research that compares the frequency, forms, contents, media, recipients, and other characteristics of leaking by later offenders and persons who showed leaking, but did not put it into action (Bondü, 2012). Research on IPH has not addressed this issue so far, but previous research on school shootings and terrorist attacks identified several criteria—different forms of leaking or specific leaking contents (Bondü, 2012; Niesse et al., 2021; Tampe & Bondü, in press)—that allowed for the reliable distinction between these two groups. For this purpose, future research should compare leaking in IPH cases with nonfatal cases in which leaking was present. Legal case files provide information from different sources, over long periods of time, as well as with regard to many different aspects and could, therefore, be used as a data source. Considering that later victims may frequently have been recipients of leaking, interviews with survivors of attempted IPH might also provide valuable information.

### Practical Implications

Although leaking is a substantial warning sign that allows for timely interventions, and although the perpetrators’ social network and the later victims often noticed leaking behavior, they did not recognize its seriousness and/or forwarded their concerns (Monckton Smith, 2020; Saxton et al., 2022; Vatnar et al., 2017). This underscores the need to encourage the reporting of leaking for example to law enforcement authorities. Tangible information about leaking may heighten the chances of its identification, widen the range of individuals that can respond to it, and increase the probability of it being reported. When informing the public about leaking, however, it should be emphasized that it is not always linked to future homicide and that an additional assessment of its seriousness is indispensable. This will guard against false reports, feelings of insecurity, as well as stereotyping and discrimination (Dudenhoefer et al., 2021).

Although many cases of IPH involved prior police contacts, structured risk assessment was rarely conducted (Hamilton et al., 2013; Koppa & Messing, 2021; Saxton et al., 2022). This highlights the need for mandatory risk assessment with efficient tools that allow one to successfully identify high-risk perpetrators, including women that currently often go unrecognized (mostly due to a strong reliance on previous violence; Graham et al., 2021). The integration of leaking into such tools may enhance their predictive validity and allow for deriving tailored interventions, such as communicating the risk toward the victim or implementing safety plans or firearm restrictions, in cooperation with social services (Horn et al., in press; Messing et al., 2015; Zeoli et al., 2018). Up to now, only some aspects of leaking have been included into risk assessment tools for IPV and/or IPH (Table 2). This includes previous (attempts of) lethal violence and nonfatal strangulation as indicators of a potential for severe violence (see above), death threats, the use of weapons, and suicidal behavior for example in the Danger Assessment (Campbell et al., 2009) or the Ontario Domestic Assault Risk Assessment (Hilton et al., 2010). The Spousal Assault Risk Assessment (SARA, Kropp & Hart, 2000) also considers homicidal ideation. However, these tools merely note the presence or absence of these forms of leaking and do not take into account their frequency, specific contents, media and development. Further potentially important forms—specifically planning activities and interests in similar offenses—have not yet been considered. Table 4 highlights implications for practice, policy and research.

**Table 2.** Items Measuring Leaking in Intimae Partner Homicide (IPH) or Intimate Partner Violence (IPV) Risk Assessment Tools.

Risk Assessment Tools	1. Homicide Announcements	2. Past Severe Violence	3. Suicidal Behavior
IPV			
ODARA	Threats to kill		
DVRAG	Threats to harm or kill		
DVSI-R	Use of weapons		
DASH	Threats to kill Use of weapons	Attempts to strangle/choke/drown	Threatened or attempted suicide
SARA	Past use of weapons and/or credible threats of death	Severe and or sexual assault	Suicidal or homicidal ideation/intent
IPH			
DA	Threats to kill Use or threat of use of weapons	Choking attempt	Threatened or attempted suicide
H-Scale			Suicide threats, ideas and attempts
IPV and IPH			
IRAD	Specific threats to kill Use or threat of use of weapons	Strangulation	Suspect suicidal
SIVIPAS	Threats with weapons		

*Notes.* Items of IPV/IPH risk assessment tools capturing leaking. Items have been assigned to categories of leaking as portrayed in Table 1. No indications for planning activities or interests in similar offenses/offenders have been found in risk assessment tools. Past physical violence has not been included, whenever its intensity was not further specified. DA: Danger Assessment (Campbell et al., 2009); DASH: Domestic Abuse, Stalking and Harassment and honor based violence (Richards, 2009); DVRAG: Domestic Violence Risk Appraisal Guide (Hilton et al., 2008); DVSI-R: Domestic Violence Screening Instrument Revised (Williams & Grant, 2006); H-Scale (López-Ossorio et al., 2021); IRAD: Idaho Risk Assessment of Dangerousness (Growette Bostaph et al., 2017); ODARA: Ontario Domestic Assault Risk Assessment (Hilton et al., 2010); SARA: Spousal Assault Risk Assessment (Kropp & Hart, 2000); SIVIPAS: Severe Intimate Violence Partner Risk Prediction Scale (Echeburúa et al., 2009).

**Table 3.** Summary of Critical Findings.

Critical Findings
• Leaking has not been systematically researched in IPH. However, publications described several behaviors by which perpetrators expressed their thoughts, feelings, fantasies, plans, or intentions to commit IPH. These behaviors demonstrate the applicability of the leaking concept to the context of IPH and were summarized into five groups, including: homicide announcements, previous severe acts of violence, suicidal behavior, planning activities, and interest in similar offenses. • Homicide announcements, including verbal expressions of homicidal intentions and threats with weapons, were the most frequently reported form of leaking and found in up to 97% of IPH cases. • Research on IPH does not capture the differentiation between threats and *leakage*, which is crucial for threat assessment. • The review identified that next to the later victims, the perpetrators’ or victims’ family, children, friends, as well as professionals in contact became recipients of leaking. • Research on IPH did not pay attention to the media by which IPH perpetrators communicate their homicidal intentions. • Subgroup differences in the perpetrators’ leaking were sparse. However, female perpetrators may more often ask for assistance in carrying out the offense than men, whereas male perpetrators may more often express their willingness to kill the victim by previous potentially lethal violence (e.g., nonfatal-strangulation) than women.

*Note.* IPH = Intimate partner homicides.

**Table 4.** Implications for Practice, Policy and Research.

Practice & Policy
• The identified forms of leaking offer an overview over potential high-risk indicators for future IPH and should be included into risk assessment tools for police officers and other frontline professionals. The usage of these tools should be mandatory. They can give guidance on when to notify law enforcement agencies, to induce further investigations, or to implement safety plans. • Police officers and other frontline professionals should be advised to share their information on leaking with each other to enable comprehensive risk assessments. • The general public should be made aware of leaking and its forms to encourage reporting of leaking to law enforcement agencies.
Research
• Future research is needed to assess leaking and its feasibility for the prevention of IPH. Other characteristics of leaking (e.g., forms or contents) should be considered to assess the seriousness of leaking, that is, the likelihood of the announcements to be put into action. • Further research is needed to examine potential differences in leaking among IPH subgroups, particularly among male and female offenders. • This review highlights the need to develop a uniform definition of leaking to ensure comparability of findings. • There is a need to examine factors and conditions under which leaking will be reported to law-enforcement agencies and to implement factors that increase reporting into primary prevention programs.

*Note.* IPH = Intimate partner homicides.

### Limitations and Outlook

When interpreting the present findings, it should be kept in mind that there has been no systematic research on leaking in cases of IPH to date, and that the relevant behaviors identified as leaking by this review were not labeled as such in the primary studies. The potential observability of the relevant behavior by (disapproving) third parties was rarely reported, calling into question whether it indeed corresponds to the definition of leaking (e.g., when conducting internet searches for murder methods; Monckton Smith, 2020). Because the studies included into this review often did not provide clear definitions of their behavioral categories and used different data sources, it is uncertain whether their findings are comparable. In addition, many publications relied on small, primarily Western, male, heterosexual, partly overlapping samples (e.g., 8 publications referred to the 11-city case-control study alone; e.g., Campbell et al., 2003; Farr, 2002; Glass et al., 2004, 2008; Koziol-McLain et al., 2006; McFarlane et al., 1999; 2002; Nicolaidis et al., 2003), calling the generalizability of their findings to other samples (e.g., female perpetrators) into question. Because the term leaking was not used in publications or abstracts, despite thorough screening, further relevant publications may have been missed. In addition, the specificity of leaking for IPH is unknown, although meta-analyses found that death threats, threats with weapons, suicidal intent, and nonfatal strangulation significantly heightened the risk for IPH but not for nonlethal-IPV (Matias et al., 2020; Spencer & Stith, 2018). Thus, future research should systematically examine leaking, its forms, contents, and other characteristics, highlight frequent recipients, and explore changes in leaking over time. Furthermore, future studies should consider additional IPH subgroups that may display leaking differently, such as offenses with victims from former and present relationships, male and female offenders, or offenses with additional victims. Because IPH has multiple causes, research and prevention efforts should not only consider leaking, but also address community and societal factors, such as the availability of housing services or firearm restrictions (Chopra et al., 2022; Graham et al., 2022; Zeoli et al., 2018). These factors might also influence the occurrence or observability of leaking. Due to the limited number of IPH studies on the time frame of the COVID-19 pandemic, we could not infer whether pandemic-related societal changes (e.g., curfew, lockdowns, and social distancing) may have influenced the occurrence, number of recipients, or observability of leaking.

## Conclusion

The current review demonstrates a lack of systematic research on leaking among IPH perpetrators. Nevertheless, numerous studies described different forms of leaking, pointing to its relevance also in this context. Further systematic research should, therefore, identify the frequency, most relevant forms, and development of leaking among IPH perpetrators. Most importantly, criteria for its likelihood of being put into action should be identified and provided to law enforcement agencies and other frontline responders to help prevent IPH.

## Supplemental Material

sj-docx-1-tva-10.1177_15248380241237213 – Supplemental material for Leaking in Intimate Partner Homicide: A Systematic Review Supplemental material, sj-docx-1-tva-10.1177_15248380241237213 for Leaking in Intimate Partner Homicide: A Systematic Review by Tanita Rumpf, Stefanie Horn, Catharina Vogt, Kristin Göbel, Thomas Görgen, Kim Marie Zibulski, Vanessa Uttenweiler and Rebecca Bondü in Trauma, Violence, & Abuse
