# Supplementary material for: Leaking in Intimate Partner Homicide: A Systematic Review
Source: Trauma Violence Abuse. 2024 Mar 29;25(4):3005–19. doi: 10.1177/15248380241237213 (PMC11370174; doi:10.1177/15248380241237213)
Supplement: sj-docx-1-tva-10.1177_15248380241237213 – Supplemental material for Leaking in Intimate Partner Homicide: A Systematic Review [file sj-docx-1-tva-10.1177_15248380241237213.docx]

**Supplementary Material A**

*Included Publications and main results on Leaking in Intimate Partner Homicides(IPH)*

|  | **Publication characteristics** | | |  | |  | **Forms of Leaking** |  |  |
| --- | --- | --- | --- | --- | --- | --- | --- | --- | --- |
| **Reference** | | ***N*** | **Subgroup** | **Data sources** | **Male %** | **Main results** | | | **%** |
| Adinkrah, 2000 | | 4 | IPH | Case files, interviews with police and other professionals, media reports | 0 | *Potentially lethal violence*: Previous attempt to kill the victim^e^ | | |  |
|  |  |  |  |  |  | *Planning activities*: Plotting the homicide^e^   Perpetrators had one or more accomplices^e^ | | | 75^d^ |
|  |  |  |  |  |  | *Recipients:* Accomplices^e^, new partner^e^ | | |  |
| Anderson et al., 2011 | | 1 | Familicide-suicide | Case file, interviews with proxy informants | 100 | *Suicidal behavior*: Disclosure of suicide thoughts and suicide plans^e^   Previous suicide attempt^e^ | | |  |
|  |  |  |  |  |  | *Recipients:* Perpetrator’s brother^e^, work colleagues^e^ | | |  |
| Boxall et al., 2022 | | 199 | IPH | Offense records, court documents supplemented with information from the National Coronial Information System and media reports | 100 | *Announcements*: Threats to kill the victim   Threats with weapons | | |  |
|  |  |  |  |  |  | *Potentially lethal violence:* Non-fatal strangulation | | |  |
|  |  |  |  |  |  | *Suicidal behavior:* Suicide thoughts and threats to attempt suicide^e^   Preparing suicide^e^ | | |  |
|  |  |  |  |  |  | *Planning activities:* Plans to murder the victim and conceal one’s own    involvement: e.g.: researching on murder methods, laying the    groundwork to establish an alibi   Purchasing or obtaining accoutrement   Taking a gun to the crime scene^e^   Offering somebody money to “bash” the victim^e^ | | |  |
|  |  |  |  |  |  | *Recipients:* Victim, former partner^e^, family (brother, children)^e^, friends^e^ | | |  |
|  |  |  |  |  |  | *Media:* Telephone messages^e^ | | |  |
| Bridger et al., 2017 | | 188 | IPH | Case files and domestic homicide reviews | 86 | *Announcements:* Disclosure of the intention of serious harm or   murder (total/men/women) | | | 20/21/12 |
|  |  |  |  |  |  | *Suicidal behavior:* Disclosure of thoughts on suicide(total/men/women)   Suicide ideation and previous suicide attempts (total/men/women) | | | 24/27/8  39/40/28 |
|  |  |  |  |  |  | *Planning activities:* Plans to dispose the victims’ body(total/men/women)   Plans to escape the country (total/men/women)   Preparation of an alibi (total/men/women)   Sourcing equipment (total/men/women) | | | 8/8/12  2/2/0  8/8/8  30/31/24 |
| Campbell et al., 2003^a^ | | 220 | IPH | Police or medical examiner reports, interviews with proxy informants | 100 | *Announcements:* Threats to kill the victim   Previous threats with weapons | | | 74  55 |
|  |  |  |  |  |  | *Potentially lethal violence:* Choking/strangulation attempt | | | 56 |
|  |  |  |  |  |  | *Suicidal behavior:* Suicide threats or attempts | | | 25 |
|  |  |  |  |  |  | *Recipients:* Victim | | |  |
| Cheng & Jaffe, 2021 | | 135 | IPH | Data provided from the Domestic Violence Death Review Committee in Ontario (DVDRC) | 100 | *Announcements:* Threats to kill the victim   Threats with weapons | | |  |
|  |  |  |  |  |  | *Potentially lethal violence*: Choking | | |  |
|  |  |  |  |  |  | *Suicidal behavior:* Threats to commit suicide   Suicide attempts | | | 60^d^  25^d^ |
|  |  |  |  |  |  | *Recipients:* Victim | | |  |
| Chopra et al., 2022 | | 263 | IPH | Domestic homicide reviews | 88 | *Announcements:* Use or threats of use of weapons | | | 16 |
|  |  |  |  |  |  | *Potentially lethal violence*: Strangulation | | | 11 |
|  |  |  |  |  |  | *Suicidal behavior*: Suicide threats or demonstration of suicide thoughts | | | 12 |
|  |  |  |  |  |  | *Recipients:* Victim | | |  |
| Cunha & Goncalves, 2016 | | 50 | (attempted)  IPH | Case files, interviews with the perpetrator, several risk assessment instruments | 100 | *Announcements:* Threats with guns | | |  |
|  |  |  |  |  |  | *Recipients:* Victim | | |  |
| Cunha & Gonçalves, 2019 | | 35 | IPH | Organizational and judicial files, interviews with the perpetrator, risk assessment instrument | 100 | *Announcements:* Use of weapons and/or credible threats of death | | | 97 |
|  |  |  |  |  |  | *Suicidal behavior.* Suicidal or homicidal ideation/intent | | | 83 |
|  |  |  |  |  |  | *Recipients:* Victim | | |  |
| David & Jaffe 2021 | | 93 | IPH (immigrant perpetrators) | DVDRC Ontario case summaries | 100 | *Announcements*: Threats with weapons | | | 43 |
|  |  |  |  |  |  | *Suicidal behavior:* Suicide threats | | | 63 |
| Enander et al., 2021 | | 50 | IPH | Court records | 80 | *Announcements:* Disclosure of intentions or plans to kill the victim   Threats of use of weapons | | |  |
|  |  |  |  |  |  | *Potentially lethal violence*: Attempts to kill the victim or a former partner^e^   Attempted strangulation^e^ | | |  |
|  |  |  |  |  |  | *Suicidal behavior:* Expression of suicide thoughts or intention   Suicide preparations^e^ | | |  |
|  |  |  |  |  |  | *Planning activities*: Homicide preparations (e.g. strategic planning)   Putting a weapon in position or arming oneself with weapons^e^ | | |  |
|  |  |  |  |  |  | *Recipients:* Victim^e^, former partner^e^, friends^e^, work colleagues^e^ | | |  |
|  |  |  |  |  |  | *Media:* Mail | | |  |
| Eriksson et al., 2022 | | *8* | IPH | Interviews with bereaved | 100 | *Announcements:* Threats to kill the victim^e^ | | |  |
|  |  |  |  |  |  | *Potentially lethal violence:* Strangulation^e^ | | |  |
|  |  |  |  |  |  | *Recipients:* Victim^e^ | | |  |
|  |  |  |  |  |  | *Media:* Private conversations^e^ | | |  |
| Farr, 2002^a^ | | 7 | IPH | Police reports and interviews with IPH survivors | 97 | *Announcements:* Threats with weapons or use of weapons^e^ | | |  |
|  |  |  |  |  |  | *Suicidal behavior:* Threats or attempts of suicide^e^ | | | 71^d^ |
|  |  |  |  |  |  | *Planning activities:* Putting a weapon in position^e^ | | |  |
|  |  |  |  |  |  | *Recipients:* Victim^e^ | | |  |
|  |  |  |  |  |  | *Media:* Gesture^e^ | | |  |
| Glass et al., 2004^a^ | | 9 | IPH in lesbian relationships | Police or medical examiner reports, interview with IPH survivors and proxy informants | 0 | *Announcements:* Threating to kill the victim or oneself   Previous use of or threat of use of weapons | | | 57  67^d^ |
|  |  |  |  |  |  | *Potentially lethal violence:* Previous attempts to kill the victim^e^   Choking | | | 44^d^ |
|  |  |  |  |  |  | *Suicidal behavior:* Suicide threats or attempts | | | 40 |
|  |  |  |  |  |  | *Planning activities:* Purchasing a gun^e^ | | |  |
|  |  |  |  |  |  | *Recipients:* Victim | | |  |
| Glass et al., 2008^a^ | | 28 | IPH (young adult IPH perpetrators) | Police reports and interviews with IPH survivors | 100 | *Announcements*: Threats to kill the victim   Threats with weapons | | | 69  30 |
|  |  |  |  |  |  | *Potentially lethal violence:* Choking | | | 35 |
|  |  |  |  |  |  | *Recipients:* Victim | | |  |
| Goussinsky & Yassour-Borochowitz, 2012 | | 18 | IPH | Interviews with IPH perpetrators | 100 | *Announcements:* Threats to kill the victim or self^e^ | | |  |
|  |  |  |  |  |  | *Suicidal behavior:* Thoughts on and threats of suicide^e^   Suicide attempts | | |  |
|  |  |  |  |  |  | *Planning activities:* Preplanned homicide   Equipping with weapons^e^ | | |  |
|  |  |  |  |  |  | *Recipients:* Victim | | |  |
| Hamilton et al., 2013 | | 84 | IPH & Familicide | DVDRC Ontario case summaries | 100 | *Announcements:* Threats to kill the victim (total/IPH/Familicide) | | | 49/46/62^d^ |
|  |  |  |  |  |  | *Suicidal behavior*: Suicide threats   Suicide attempts | | | 50/49/54^d^  24/23/31^d^ |
| Hesselink & Dastile, 2015 | | 15 | IPH | Interviews with IPH perpetrators | 0 | *Planning activities:* Instruction or hiring of a third person^e^ | | | 67^d^ |
|  |  |  |  |  |  | *Recipients:* Friends, professionals^e^ | | |  |
| Jaffe et al., 2014 | | 40 | IPH & Familicide | DVDRC Ontario case summaries, risk assessment instruments | 100 | *Announcements:* Death threats (IPH/Familicide)   Threats with a lethal weapon in (IPH/Familicide) | | | 63/69  30/31 |
|  |  |  |  |  |  | *Potentially lethal violence:* Choking attempt (IPH/Familicide) | | | 15/22 |
|  |  |  |  |  |  | *Suicidal behavior*: Threats or attempts of suicide (IPH/Familicide) | | | 48/54 |
|  |  |  |  |  |  | *Recipients:* Victim | | |  |
| Kapardis et al., 2017 | | 8 | IPH | Interviews with IPH orphans | 100 | *Announcements:* Threats to kill the victim^e^ | | |  |
|  |  |  |  |  |  | *Potentially lethal violence:* Previous attempts to kill the victim (by strangulation^e^) | | | 25^d^ |
|  |  |  |  |  |  | *Planning activities:* Premeditation | | |  |
|  |  |  |  |  |  | *Recipients:* Victim^e^, children^e^ | | |  |
| Koziol-McLain et al., 2006^a^ | | 219 | IPH & IPH-suicide | Police or medical examiner reports, interviews with proxy informants | 100 | *Announcements:* Threats to kill the victim    (total/IPH without suicide/IPH-suicide)   Threats with weapons (total/IPH without suicide/IPH-suicide) | | | 73^d^/71/78  56^d^/56/56 |
|  |  |  |  |  |  | *Potentially lethal violence:* Choking attempt    (total/IPH without suicide/IPH-suicide) | | | 56^d^/58/52 |
|  |  |  |  |  |  | *Suicidal behavior:* Suicide threats    (total/ IPH without suicide /IPH-suicide) | | | 25^d^/14/51 |
|  |  |  |  |  |  | *Recipients:* Victim | | |  |
| Leygraf, 2015^c^ | | 71 | IPH | Forensic reports | 82 | *Planning activities:* Homicide plans (total/male/female)   Arming oneself with weapons^e^   Asking for assistance^e^ | | | 54^d^/50/69 |
|  |  |  |  |  |  | *Recipients:* Accomplices^e^ | | |  |
|  |  |  |  |  |  | *Media:* Written plans | | |  |
| Logan et al., 2013 | | 30 | Familicide-suicide | Coroner and medical examiner reports, toxicology reports, law enforcement records, death certificates | 90 | *Suicidal behavior:* Disclosure of suicidal intentions   Suicide Notes | | | 13  30 |
|  |  |  |  |  |  | *Media:* Notes | | |  |
|  |  |  |  |  |  | *Recipients:* Work colleagues, family, friends | | |  |
| López-Ossorio et al., 2021 | | 159 | IPH | National database and police reports | 100 | *Announcements:* Threats with a cutting weapon, or a firearm | | |  |
|  |  |  |  |  |  | *Suicidal behavior:* Expression of suicidal ideas or suicide threats   Suicide attempts | | |  |
|  |  |  |  |  |  | *Recipients:* Victim | | |  |
| McFarlane et al., 1999^a^ | | 206 | IPH | Police or medical examiner reports, interviews with proxy informants or IPH survivors | NA | *Announcements:* Frightening the victim with a weapon    (IPH/attempted IPH) | | | 39/40 |
|  |  |  |  |  |  | *Suicidal behavior:* Suicide threats (IPH/attempted IPH) | | | 19/34 |
|  |  |  |  |  |  | *Recipients:* Victim | | |  |
| McFarlane et al., 2002^a^ | | 437 | IPH | Police reports, interviews with proxy informants or IPH survivors | NA | *Announcements*: Threats to kill the victim   Frightening the victim with a weapon | | | 55  40 |
|  |  |  |  |  |  | *Recipients:* Victim | | |  |
| Moen et al., 2016 | | 65 | IPH | Court judgements | 0 | *Planning activities:* Conscious planning of murder   Having accomplices | | | 15 |
|  |  |  |  |  |  | *Recipients:* Accomplices | | |  |
| Monckton Smith, 2016 | | 1 | IPH | Individual Manage Reviews provided by involved agencies, statements from police officers, witnesses and the victim, interview with the victim’s GP | 100 | *Announcements:* Threats to the victim’s life^e^ | | |  |
|  |  |  |  |  |  | *Potentially lethal violence:* Attempted strangulation^e^ | | |  |
|  |  |  |  |  |  | *Recipients:* Victim^e^, neighbor^e^ | | |  |
| Monckton Smith, 2020 | | 372 | IPH | Domestic homicide reviews, documentaries, court reportings, transcripts of parliamentary proceedings, news and media reports, professional, perpetrator and family reflections | 100 | *Announcements:* Disclosure of plans to kill the victim, threats to kill the    victim^e^ | | |  |
|  |  |  |  |  |  | *Potentially lethal violence:* Attempted Strangulation^e^ | | |  |
|  |  |  |  |  |  | *Suicidal behavior:* Suicide threats | | |  |
|  |  |  |  |  |  | *Planning activities:* Homicide plans (e.g. researching on methods to kill,    conducting plans to conceal the victim’s body)   Purchase of weapons   Putting a weapon in position | | |  |
|  |  |  |  |  |  | *Recipients:* Victim, former partner, police^e^ | | |  |
|  |  |  |  |  |  | *Media:* Written plans, internet search engines^e^ | | |  |
| Musielak et al., 2019 | | 183 | IPH | Data provided by the DVDRC in Ontario and case files | 100 | *Announcements:* Threats to kill the victim | | |  |
|  |  |  |  |  |  | *Suicidal behavior:* Suicide threats | | |  |
| Nicolaidis et al., 2003^a^ | | 30 | IPH | Interviews with survivors of attempted IPH supplemented with Danger Assessment scores | 100 | *Announcements:* Threats to kill the victim   Threats with guns^e^ | | |  |
|  |  |  |  |  |  | *Potentially lethal violence:* Attempts to kill a former partner^e^ | | |  |
|  |  |  |  |  |  | *Recipients:* Victim, former partner^e^ | | |  |
| Pontedeira et al., 2020 | | 24 | IPH | Court sentences | 92 | *Announcements:* Threats to kill the victim | | | 63 |
|  |  |  |  |  |  | *Potentially lethal violence:* Previous attempts to kill the victim | | | 25 |
|  |  |  |  |  |  | *Suicidal behavior:* Previous suicide attempts or ideas | | | 4^d^ |
|  |  |  |  |  |  | *Planning activities:* Premeditation, e.g. taking the weapon to the crime    scene | | | 42^d^ |
|  |  |  |  |  |  | *Recipients:* Victim | | |  |
| Pottinger et al., 2019 | | 27 | IPH-Suicide | Police reports | 100 | *Suicidal behavior:* Suicide notes | | |  |
|  |  |  |  |  |  | *Media:* Note | | |  |
| Rabe & Heubrock, 2013 | | 1 | IPH | Court judgements, interview with proxy informants, media reports | 100 | *Announcements*: Disclosure of thoughts and intentions to kill the victim^e^ | | |  |
|  |  |  |  |  |  | *Planning activities:* Purchase of homicide-equipment^e^   Asking for assistance or a contract killer^e^ | | |  |
|  |  |  |  |  |  | *Recipients:* Family (uncle and his girlfriend) ^e^, acquaintance^e^ | | |  |
| Regan et al., 2007 | | 7 | IPH | Case files and interviews with the perpetrator’s informal network and agency informants | 100 | *Announcements:* Threats to kill the victim   Use of or threats with weapons | | | 29^d^  29^d^ |
|  |  |  |  |  |  | *Potentially lethal violence:* Attempted strangulation^e^ | | |  |
|  |  |  |  |  |  | *Suicidal behavior:* Threats of or preoccupation with suicide   Suicide preparations^e^   Attempted suicide^e^ | | | 71^d^ |
|  |  |  |  |  |  | *Interest in similar offenses/offenders:* Conversation with friends about    “wife-killing”^e^ | | |  |
|  |  |  |  |  |  | *Recipients:* Victim^e^, family ^e^(children, brother) ^e^, friends^e^,    work colleagues^e^, psychiatrist^e^, formal networks^e^ | | |  |
| Rye & Angel, 2019 | | 77 | IPH | National database information, case files, criminal records | 84 | *Announcements:* Disclosure of thoughts on serious harm or murder    (total/men/women)   Threat to life either against the partner or others (total) | | | 32/31/33  55 |
|  |  |  |  |  |  | *Potentially lethal violence*: Choking or strangulation (men) | | | 25 |
|  |  |  |  |  |  | *Suicidal behavior:* Thoughts on, threats of and attempts of suicide    (total/men/women) | | | 53/52/58 |
|  |  |  |  |  |  | *Planning activities:* Preparation of the homicide (total/female) | | | 40/25 |
|  |  |  |  |  |  | *Recipients:* Victim | | |  |
| Saxton et al., 2022 | | 176 | IPH | Data provided from the DVDRC in Ontario | 100 | *Announcements:* Threats to kill the victim   Threats with a weapon | | | 44^d^  26^d^ |
|  |  |  |  |  |  | *Potentially lethal violence:* Choking | | | 16^d^ |
|  |  |  |  |  |  | *Suicidal behavior:* Suicide threats or attempts | | | 51^d^ |
|  |  |  |  |  |  | *Recipients:* Victim | | |  |
| Sharp-Jeffs & Kelly, 2016 | | 24 | IPH | Domestic homicide reviews | 96 | *Announcements:* Threats to kill the victim   Use of or threat of use of weapons | | | 29^d^  21^d^ |
|  |  |  |  |  |  | *Potentially lethal violence*: Strangulation attempt | | | 17^d^ |
|  |  |  |  |  |  | *Suicidal behaviors:* Previous thoughts on, threats or attempts of suicide | | | 29^d^ |
|  |  |  |  |  |  | *Recipients:* Victim^e^, statutory agencies, general practitioners,    psychiatrist^e^, police^e^ | | |  |
|  |  |  |  |  |  | *Media:* Phone call^e^ | | |  |
| Sheehan et al., 2014 | | 9 | IPH | Affidavits, media reports and interviews with bereaved | 78 | *Announcements:* Disclosure of homicide plans   Previous threat with a weapon^e^ | | |  |
|  |  |  |  |  |  | *Potentially lethal violence:* Strangulation attempt^e^ | | |  |
|  |  |  |  |  |  | *Planning activities:* Purchase of weapons^e^   Arming oneself with weapons^e^ | | |  |
|  |  |  |  |  |  | *Recipients:* Victim^e^, friends^e^, victim’s father^e^ | | |  |
|  |  |  |  |  |  | *Media:* Gesture^e^ | | |  |
| Steck, 2005^b^ | | *51* | IPH | Interview with perpetrators | 63 | *Announcements:* Disclosure of the intention to kill the victim(men/women) | | | 22/47^d^ |
|  |  |  |  |  |  | *Suicidal behavior:* Suicide threats (only reported for men) | | |  |
|  |  |  |  |  |  | *Planning activities:* Arming oneself with weapons or engagement of an accomplice (men/women) | | | 9/47^d^ |
|  |  |  |  |  |  | *Recipients:* Accomplices | | |  |
| Taylor, 2009 | | 57 | IPH | Chicago Homicide dataset, medical examiner and criminal justice records and media reports | 100 | *Announcements:* Death threats | | | 44 |
| Todd et al., 2020 | | 41 | IPH | Domestic Homicide Reviews and interviews with IPH survivors or bereaved | 81 | *Announcements:* Threats to kill the victim^e^, homicide announcements^e^ | | |  |
|  |  |  |  |  |  | *Suicidal behavior:* Threats to commit suicide^e^ | | |  |
|  |  |  |  |  |  | *Recipients:* Victim^e^ | | |  |
|  |  |  |  |  |  | *Media:* Phone calls, text messages^e^ | | |  |
| Toprak & Ersoy, 2017 | | 79 | IPH | Police and medical examiner reports | 100 | *Announcements:* Previous or immediate threats to kill the victim | | | 26 |
| Vatnar et al., 2017; 2019 | | 177 | IPH & IPH-suicide | Court documents and interviews with bereaved | 89 | *Announcements:* Threats to kill the victim    (total/IPH without suicide/IPH-suicide)   Disclosure of plans to kill the victim    (total/IPH without suicide/IPH-suicide) | | | 54/60/36  40/40/39^d^ |
|  |  |  |  |  |  | *Suicide behavior:* Suicide ideation and suicide threats | | |  |
|  |  |  |  |  |  | *Recipients:* Friends, Family | | |  |
|  |  |  |  |  |  | *Media:* Private conversations | | |  |
| Weeke & Oberwittler, 2017 | | ≈ 220 | IPH- & Familicide-Suicide | Prosecution files | NA | *Suicidal behavior:* Suicide Notes (total/IPH/Familicide) | | | 39^d^/40/36 |
|  |  |  |  |  |  | *Media:* Notes | | |  |
| Wiltsey, 2008 | | 32 | IPH | Legal case files | 100 | *Announcements:* Prior threats to kill the victim   Threats with weapons | | | 41  19 |
|  |  |  |  |  |  | *Recipients:* Victim | | |  |

*Notes.^.^* Forms of Leaking have been grouped according to those portrayed in Table 1. Whenever mentioned, frequencies for IPH subgroups (male vs. female, IPH-suicide, familicide) were reported separately. Frequencies are based upon those reported within studies.

^a^Publications used data from the 11-cities case-control study that compares risk factors between IPH and IPV perpetrators.

^b^Steck (2002) described and compared two datasets whose results will be summarized. The first dataset analyzed 32 male and the second 19 female IPH perpetrators.

^c^The original sample additionally included 13 IPH perpetrators that were found not guilty by reason of insanity (NGRI). Reported information excluded reported results of the sample additionally including NGRI-offenders.

^d^Frequencies have been calculated based upon information contained within publications.

^e^Information is based on case example
